# Vertical and Horizontal Trust at Work as Predictors of Retirement Intentions: The Finnish Public Sector Study

**DOI:** 10.1371/journal.pone.0106956

**Published:** 2014-09-05

**Authors:** Charlotte Muurinen, Matti Laine, Jaana Pentti, Marianna Virtanen, Paula Salo, Mika Kivimäki, Jussi Vahtera, Tuula Oksanen

**Affiliations:** 1 Finnish Institute of Occupational Health, Development of Work and Organisations, Turku and Helsinki, Finland; 2 Åbo Akademi University, Department of Psychology and Logopedics, Turku, Finland; 3 University of Turku, Department of Behavioral Science and Philosophy, Psychology section, Turku, Finland; 4 University College London, Department of Epidemiology and Public Health, London, United Kingdom, and University of Helsinki, Institute of Behavioral Sciences, Helsinki, Finland; 5 University of Turku, Department of Public Health, Turku, and Turku University Hospital, Turku, Finland; George Mason University/Krasnow Institute for Advanced Study, United States of America

## Abstract

This prospective cohort study aimed to examine the associations of trust towards the supervisor (vertical trust) and trust towards co-workers (horizontal trust) with retirement intentions. The participants were 14 840 women and men working in the municipal sector in 2000–12 (Finnish Public Sector Study). Trust (vertical trust towards the supervisor and horizontal trust towards co-workers) and retirement intentions were assessed in repeated surveys. Multivariable multinomial logistic regression analyses were conducted to assess the association between baseline trust and retirement intentions at 3.7 years of follow-up. Demographic characteristics, health, psychological distress, health risk behaviors, personality factors, and psychosocial factors were included as covariates. Of the participants, 67.0% trusted their supervisor and 54.9% trusted their co-workers. Employees who trusted their supervisor (odds ratio (OR) 0.60, 95% confidence interval (CI) 0.53–0.67) and employees who trusted their co-workers (odds ratio 0.62, 95% confidence interval 0.55–0.70) at baseline were less likely to have strong retirement intentions at follow-up compared to those who did not trust. These associations largely persisted after adjusting for all covariates and taking into account baseline retirement intentions. In conclusion, trust in the supervisor and co-workers predicted retirement intentions. These observational findings suggest that increasing trust in the workplace may contribute to lengthening working careers and preventing early retirement.

## Introduction

In most high-income countries the population is ageing: life expectancy is increasing, while birth rates are decreasing [1], [2]. According to demographic projections, there will be a shortage of qualified labor force challenging the pension systems and the sustainability of public finance [3]. Identifying and intervening in the factors that contribute to the process of early retirement is highly important to lengthen working careers and postpone or prevent early retirement.

The process of early retirement is influenced by a wide range of both work and non-work related factors [4], [5], [6]. These factors are often divided into factors that push the person to leave the workforce, such as poor health, and factors that pull the person towards retirement, such as a retired spouse [5], [7]. Importantly, employees’ intentions to retire early strongly predict actual retirement [4], [8]. Thus, the process of early retirement can be seen as a result of the interplay between both personal and environmental factors [4].

People with sufficient economic resources and poor health are more likely to retire early [9]. Also health-related risk behaviors, such as excess alcohol consumption, are associated with early exit from the labor market [10]. Low socioeconomic status has been shown to relate to early retirement intentions [11], but these findings are inconsistent [5]: higher paid employees are more likely to retire early than lower paid employees [12]. Other important factors are older age [6], poor work ability [13], familial factors, such as marital, dependent care, and spouse’s working status [6], and the pension system of the country (e.g. public or private early retirement schemes) [14].

Several psychosocial factors at work, such as job strain [15], effort-reward imbalance [11], poor job satisfaction [12], [16], poor work motivation [17], and low organizational commitment [13] have also been found to be associated with intentions to retire and with early retirement. On the other hand, good quality of the near superior’s management [18] and perceived fairness in supervision [19] have been associated with weaker retirement intentions. Since fairness, justice and trust are partially overlapping concepts [20] it is possible that trust could also affect retirement thoughts.

Results from studies in different disciplines suggest that trust is highly beneficial for organizations [20], [21]. Trust in the workplace has been found to be associated with various beneficial attitudinal and performance outcomes, including job satisfaction, decreased turnover intent, higher organizational commitment [21], [22], favorable organizational citizenship behaviors, improved performance [21], [22], [23], [24], and the effectiveness of human resource management practices [25]. Mistrust at work, in turn, has been linked to poor health [26]. However, the role of trust in retirement intentions is not known.

The aim of the present study was to investigate the longitudinal associations of trust towards the supervisor (vertical trust) and trust towards co-workers (horizontal trust) with retirement intentions in a large cohort of public sector employees. Our hypothesis was that trust in the supervisor and in co-workers is associated with lower probability of having retirement intentions at follow-up.

## Methods

### Ethics statement

The study was approved by the Ethics Committee of Helsinki-Uusimaa Hospital District. The response to a questionnaire acted as a form of written informed consent. All data were analyzed anonymously.

### Study Context

This study was conducted in Finland, where since 2005 the national old-age pension for both men and women is payable from age 65, but age-based retirement is possible between the ages of 63 and 68 [27]. During this window period, the pension accrual rises sharply. In some cases, the pensionable age can be lower than 63, for example depending on the occupation (e.g. fire-fighters) and the employee’s job entry year. At the age of 60/61, an employee may also choose a voluntary part-time pension which is not disability-based. A disability pension may be granted if, due to an illness or injury, the employee cannot return to work within 300 workdays of reimbursed sickness absence during two consecutive years, and the disability is deemed to be long-term or permanent [28]. In 2012, the average exit age from the labor force was 61.9 for women and 61.8 for men [29].

Our study also includes data collected during the old pension scheme, before 2005. The old pension scheme included a normal retirement age of 65 and the possibility to retire earlier between ages 60 and 64 [30]. There were also many different pathways into retirement, including an unemployment pension, several variants of disability pensions, and the part-time pension. The reform in 2005 have abolished several pathways and made others financially not attractive.

### Study sample

Data were obtained from the Finnish Public Sector Study, an ongoing prospective study exploring behavioral and psychosocial factors and health in a large cohort of public sector employees working in 10 towns and 21 hospitals. The sample covers almost 20% of the Finnish municipal sector employees.

In this study, we used data from municipal employees of ten towns participating in the Finnish Public Sector Study collected in 2000–01 (N = 32 299, response rate 67%), 2004 (N = 32 197, response rate 65%), 2008 (N = 38 814, response rate 70%), and 2012 (N = 39 194, response rate 69%). The total number of participants who responded to at least one survey was 72 449. We only included 35 883 participants who had follow-up data; that is they had participated in two consecutive surveys: in 2000–01 and 2004, 2004 and 2008, or 2008 and 2012 (three potential follow-up periods). If the respondent had data on more than one period, we used data from the earliest. Of them, we included 18 198 participants who were older than 45 years at baseline, since we did not consider it relevant to study younger people’s retirement intentions. Furthermore, respondents with missing data in any of the study variables (n = 2 696) and those who reported having already applied for a pension at follow-up (n = 662) were excluded. Thus, the final sample consisted of 14 840 participants (3 362 men and 11 478 women) aged 45–65 (mean age 50.8) at baseline.

### Trust

Direct measures of trust, that is measures that simply ask people to rate the extent to which they trust other people, have been used in earlier studies [31], [32]. In the present study, we used two items to measure trust in the workplace: one measuring trust in the supervisor (vertical trust) and another trust in the co-workers (horizontal trust). The participants were asked to indicate how much they agreed with the following statements: “We can trust our supervisor”, indicating vertical trust, and “We can really trust people in our workplace”, referring to horizontal trust. The response options were: “I strongly agree”, “I somewhat agree”, “I neither agree nor disagree”, “I somewhat disagree”, or “I strongly disagree”. Following earlier literature that dichotomizes trust (trust and mistrust/no trust) [26], [33], [34], the responses were dichotomized, so that the response options “I strongly agree” and “I somewhat agree” indicated trust, while the other response options (“I neither agree nor disagree”, “I somewhat disagree”, or “I strongly disagree”) indicated no trust.

### Intentions to retire

Retirement intentions at follow-up were measured with the following question: “Have you considered seeking disability pension, individual early retirement pension or any other form of pension?” [35]. The response options were: “I have not considered”, “It has crossed my mind”, “I have seriously considered seeking pension”, and “I have already applied for pension”. This question has been used in several Finnish studies [35]. As previously [8], intentions to retire early were divided into three categories: 1 = no intentions to retire early, 2 = weak intentions, 3 = strong intentions. The fourth category, “I have already applied for pension”, was excluded, since a pension application, instead of expressing an intention, suggests that a decision already has been made [8].

### Baseline characteristics

Demographic characteristics, including the participants’ age, gender, and socioeconomic status (SES), were obtained from employers’ registers. SES was based on the occupational-title classification of Statistics Finland: high (upper-grade non-manual workers, e.g. teachers), intermediate (lower-grade non-manual workers, e.g. registered nurses), and low (manual workers, e.g. cleaners) [36]. Marital status (married or cohabiting vs. other) and self-rated health (very good or good vs. less) was obtained from survey responses, as well as psychological distress, measured by a 12-item version of the General Health Questionnaire and dichotomized as scores ≥4 (psychological distress) vs. less [37], [38]. The health risk behaviors were assessed with standard survey measures and included current smoking status (smoker vs. non-smoker), excess alcohol consumption (average weekly consumption >210 g of absolute alcohol vs. less [39]), obesity (body mass index, BMI, from self-reports of height and weight ≥30 kg/m^2^ vs. less [40]), and leisure time physical activity (≥2.0 metabolic equivalent task [MET] hours per week, corresponding approximately to 30 min of walking per day vs. less [41]).

Since personality may be related to retirement intentions [42] and trust [24], [43], we also included two personality measures: trait anxiety and dispositional optimism. Trait anxiety was assessed in the survey using six items from the Trait Anxiety Inventory (Cronbach α = 0.85) [44]. A sum score was calculated for each participant, with higher scores referring to greater anxiety. Dispositional optimism was measured using a structured survey tool, the revised Life Orientation Test (LOT-R) (Cronbach α = .82) [45]. A sum score for optimism was calculated for each participant, with higher scores referring to higher optimism.

We obtained job strain and effort-reward imbalance (ERI) from the surveys. We did not include relational justice, that is perceived fairness in the interpersonal treatment of employees by their supervisors, because the concept includes trust in the supervisor [46]. Hence, controlling for relational justice would have led to overadjustment. Job strain was assessed using three items measuring job demands (Cronbach α = 0.77) and nine items measuring job control (Cronbach α = 0.82), derived from the Job Content Questionnaire [47]. The job strain score for each participant was formed by subtracting the mean of job control scores from the mean of job demand scores. A higher score indicated greater job strain. ERI was assessed using a proxy measure of ERI including one question about effort in work and three questions about rewards (Cronbach α = 0.64). The ERI score was obtained by calculating the ratio between the response score in the effort scale and the mean response score in the reward scale [48]. Larger values indicated larger imbalance.

### Statistical analysis

General characteristics of the study population were analyzed using descriptive statistics. We conducted multivariable multinomial logistic regression analyses using generalized logit model to examine whether trust in co-workers and trust in the supervisor were associated with intentions to retire early (categorized as no intentions, weak, or strong intentions) at follow-up. The results are presented as odds ratios (OR) and their corresponding 95% confidence intervals (95% CI).

The contribution of covariates to the associations between trust and retirement intentions was examined by including them stepwise in the models. After the crude model, in model 1, analyses were conducted for both independent variables (vertical and horizontal trust), adjusting for age, gender, SES, and marital status at baseline. Thereafter, in model 2, baseline self-rated health and psychological distress were added to the model. In model 3, the baseline health risk behaviors (current smoking status, alcohol consumption, obesity, and leisure time physical activity) were added. In model 4, baseline trait anxiety and dispositional optimism were added. Finally, model 5 also incorporated baseline job strain and ERI (full model).

To test the direction of the association, we conducted sensitivity analyses including only those participants who at baseline had expressed that they had no retirement intentions (n = 11 566).

Because age at baseline could influence retirement intentions, we additionally run a sensitivity analysis including only participants between 50–60 years of age at baseline.

All statistical analyses were performed using the SAS© 9.2 statistical software (SAS Institute Inc., Cary, NC, USA).

## Results

### Descriptive information

The descriptive characteristics and the proportions of trust in the study population at baseline are displayed in **Table 1**. The majority (77.4%) were women and 45.0% from the intermediate SES group. In the study population, 67.0% reported that they trust their supervisor. In the study population, 54.9% reported that they really trust their co-workers. Of the participants, 44.7% reported that they both trust their supervisor and really trust their co-workers. Women were more likely than men to trust co-workers, while no difference between men and women were found in trust towards the supervisor. Participants who represented the high SES group reported more trust than those in the lower groups. Moreover, participants with good self-rated health and no psychological distress reported significantly more often trust than their counterparts.

**Table 1:** The descriptive characteristics and the proportion of trust in the study population at baseline.

	N	%	Trust in supervisor			Trust in co-workers		
			N	%^a^	p for difference^b^	N	%^a^	p for difference^b^
**Number of participants**	14 840	100	9943	67.0		8146	54.9	
**Sex**					p = .109			p = .047
Male	3362	22.7	2291	68.1		1795	53.4	
Female	11478	77.4	7652	66.7		6351	55.3	
**Socioeconomic status**					p<.0001			p<.0001
Upper non-manual	5477	36.9	3789	69.2		3336	60.9	
Lower non-manual	6683	45.0	4365	65.3		3509	52.5	
Manual	2680	18.1	1789	66.8		1301	48.5	
**Marital status**					p = .005			p = .0007
Single, divorced, widowed	3536	23.8	2300	65.1		1853	52.4	
Married or cohabiting	11304	76.2	7643	67.6		6293	55.7	
**Self-rated health status**					p<.0001			p<.0001
Very good or good	10373	69.9	7321	70.6		6090	58.7	
Average or worse	4467	30.1	2622	58.7		2056	46.0	
**Psychological distress** c					p<.0001			p<.0001
No	11259	75.9	7939	70.5		6637	59.0	
Yes	3581	24.1	2004	56.0		1509	42.1	
**Health risk behavior**								
**Current smoker**					p = .0002			p<.0001
No	12194	82.2	8253	67.7		6802	55.8	
Yes	2646	17.8	1690	63.9		1344	50.8	
**Heavy drinker** d					p = .441			p = .223
No	13248	89.3	8890	67.1		7295	55.1	
Yes	1592	10.7	1053	66.1		851	53.5	
**Physically inactive** e					p = .001			p<.0001
No	11052	74.5	7487	67.7		6174	55.9	
Yes	3788	25.5	2456	64.8		1972	52.1	
**Obese (BMI≥30** **kg/m^2^)**					p = .003			p<.0001
No	12683	85.5	8558	67.5		7048	55.6	
Yes	2157	14.5	1385	64.2		1098	50.9	

a Percentages are row percentages.

b Based on chi-square test.

c Individuals scoring ≥4 using 12-item version of the General Health Questionnaire are classified as psychologically distressed.

d Average weekly consumption >210 g of absolute alcohol = heavy drinker.

e Leisure time physical activity <2 metabolic equivalent task (MET) per week = physically inactive.

The mean follow-up time was 3.7 years. At follow-up, the majority of the study population (70.1%) had no intentions to retire, 21.6% had weak intentions to retire, and 8.4% had strong intentions to retire.

### Associations between trust and retirement intentions

Vertical trust, i.e. trust in the supervisor, was associated with a lower likelihood of retirement intentions (**Table 2**). People who trusted their supervisor were less likely than their counterparts to have strong retirement intentions (vs. no intentions) (OR 0.60, 95% CI 0.53–0.67), or weak retirement intentions (vs. no intentions) (OR 0.74, 95% CI 0.68–0.80) at follow-up. Adjusting for health-related covariates attenuated the associations slightly. The results remained statistically significant after adjustment for all the covariates in the multivariable adjusted model, for strong vs. no retirement intentions (OR 0.74, 95% CI 0.65–0.85) and weak vs. no retirement intentions (OR 0.85, 95% CI 0.78–0.94).

**Table 2:** Associations between trust in the supervisor and subsequent retirement intentions: odds ratios (OR) and their 95% confidence intervals (95% CI) from multivariable multinomial logistic regression models.

	Weak vs. no intentions		Strong vs. no intentions	
	OR	95% CI	OR	95% CI
**Model** a				
Crude	0.74	0.68–0.80	0.60	0.53–0.67
Model 1^a^	0.71	0.65–0.78	0.55	0.49–0.63
Model 2^b^	0.80	0.73–0.87	0.65	0.58–0.74
Model 3^c^	0.80	0.74–0.88	0.66	0.58–0.75
Model 4^d^	0.83	0.76–0.90	0.67	0.59–0.77
Model 5^e^	0.85	0.78–0.94	0.74	0.65–0.85

a Includes demographic characteristics (age, sex, socioeconomic status, and marital status).

b Includes demographic characteristics, self-rated health, and psychological distress.

c Includes demographic characteristics, self-rated health, psychological distress, and health risk behaviors (current smoking status, alcohol consumption, obesity, and leisure time physical activity).

d Includes demographic characteristics, self-rated health, psychological distress, health risk behaviors, and personality factors (trait anxiety and dispositional optimism) (full model).

e Includes demographic characteristics, self-rated health, psychological distress, health risk behaviors, personality factors, and psychosocial factors (job strain and effort-reward imbalance) (full model).

Horizontal trust, i.e. trust in co-workers, was associated with a lower likelihood of retirement intentions (**Table 3**). People who reported that they really trust their co-workers were less likely than their counterparts to have strong retirement intentions (vs. no intentions) (OR 0.62, 95% CI 0.55–0.70), or weak retirement intentions (vs. no intentions) (OR 0.72, 95% CI 0.66–0.78) at follow-up. Adjusting for health-related covariates attenuated the associations slightly. The results also remained statistically significant after adjustment for all the covariates in the multivariable adjusted model, for strong vs. no retirement intentions (OR 0.77, 95% CI 0.68–0.88) and weak vs. no retirement intentions (OR 0.85, 95% CI 0.78–0.93).

**Table 3:** Associations between trust in co-workers and subsequent retirement intentions: odds ratios (OR) and their 95% confidence intervals (95% CI) from multivariable multinomial logistic regression models.

	Weak vs. no intentions		Strong vs. no intentions	
	OR	95% CI	OR	95% CI
**Model**				
Crude	0.72	0.66–0.78	0.62	0.55–0.70
Model 1^a^	0.71	0.65–0.77	0.58	0.52–0.66
Model 2^b^	0.79	0.73–0.86	0.68	0.60–0.78
Model 3^c^	0.80	0.73–0.87	0.69	0.61–0.78
Model 4^d^	0.83	0.76–0.90	0.71	0.63–0.81
Model 5^e^	0.85	0.78–0.93	0.77	0.68–0.88

a Includes demographic characteristics (age, sex, socioeconomic status, and marital status).

b Includes demographic characteristics, self-rated health, and psychological distress.

c Includes demographic characteristics, self-rated health, psychological distress, and health risk behaviors (current smoking status, alcohol consumption, obesity, and leisure time physical activity).

d Includes demographic characteristics, self-rated health, psychological distress, health risk behaviors, and personality factors (trait anxiety and dispositional optimism) (full model).

e Includes demographic characteristics, self-rated health, psychological distress, health risk behaviors, personality factors, and psychosocial factors (job strain and effort-reward imbalance) (full model).

In sensitivity analyses, including only participants who at baseline had no retirement intentions, we observed similar results. People who trusted their supervisor were less likely than their counterparts to have retirement intentions at follow-up (model 5, strong vs. no intentions: OR 0.86, 95% CI 0.69–1.06; model 5, weak vs. no intentions: OR 0.90, 95% CI 0.81–1.01). People who reported that they really trust people at work were less likely than their counterparts to have retirement intentions at follow-up (OR 0.86, 95% CI 0.70–1.06 and OR 0.89, 95% CI 0.80–0.99, respectively).

The results from sensitivity analyses including only participants between 50–60 years of age at baseline were consistent with the main results. (Data not shown).

## Discussion

In this large cohort study of public sector employees, we found trust in the supervisor and trust in co-workers to be associated with lower likelihood to express intentions to retire at follow-up. Employees who trusted their supervisors at baseline were 26–40% less likely to report retirement intentions at follow-up than those who did not trust their supervisor. Similarly, employees who trusted their co-workers had 28–38% lower likelihood to express intentions to retire at follow-up. Our findings largely persisted after controlling for demographic characteristics, health, personality factors, and psychosocial factors, and after excluding those with baseline retirement intentions.

Our findings are in accordance with previous research that has found that psychosocial factors at work, such as the quality of the management [18], [19], may be associated with intentions to retire [11]. Our study extends previous studies that have found trust to be associated with work attitudes, turnover intent, and organizational commitment [21], [22], by showing in a prospective study setting that trust in the workplace might also play a role in the development of retirement intentions.

Several studies draw a distinction between vertical and horizontal trust, that is trust in leaders and trust in co-workers [22]. Some researchers suggest that needs vary by group of people [20], for example, employees may have different needs towards the supervisor than towards co-workers. These needs determine how vulnerable the employees are in the relationship [22], and, therefore, it is possible that trust in different trust referents (i.e. trustees, here supervisor or co-workers) can vary in importance. Supervisors have been found to be particularly important, since they have the authority to make important decisions for the subordinate [22]. However, some studies have found that the trust referent has little influence on the magnitude of the associations with antecedents and outcomes [24]. In the present study, we found that more employees trusted their supervisor than their co-workers; nevertheless, there was no difference in the associations with retirement intentions.

Health is an important factor in the process of early retirement, and in line with previous research [9], [26], health related factors were associated with both trust and retirement intentions. On the one hand, health could mediate the association between trust at work and retirement intentions, that is low trust is associated with poor health, which in turn is related to needs to retire and stronger retirement intentions. On the other hand, health may be a prior common cause of both trust and retirement intentions. However, health alone is unlikely to explain the associations found in this study, since our results only slightly attenuated when controlling for baseline health.

Another possible explanation for the observed associations is that the distress that may follow from low levels of trust affect the employee’s attitudes towards the workplace [22]. According to a conceptual model by Mayer, Davis, and Schoorman [49], trust is “willingness to be vulnerable to another party” and leads to increased risk taking with the trustee [24], [49]. The employee with low trust might consider leaving because of concern about the decisions that the leader might make and the risks of staying [21], [50]. This has been hypothesized to explain associations with turnover intentions, but the same could be possible for retirement intentions.

According to the Social Exchange Theory [51], successful social exchange requires trust. When the employee trusts the supervisor, he or she is likely to reciprocate and respond with positive work attitudes through increased motivation and commitment. In earlier studies, work motivation has been found to be associated with willingness to work beyond retirement age [17]. Another possible explanation could be job satisfaction: people who trust their supervisor and co-workers might feel more satisfied at work [21], [50], which in turn explains weaker retirement intentions [12]. More research is needed to explore the possible mechanisms underlying the associations between trust and retirement intentions.

### Strengths and limitations

To our knowledge, this is the first study focusing on the association between trust and retirement intentions. The strengths of this study include a large sample size, high response rate, prospective study design, and statistical control for a number of potential and already known confounding factors. However, some methodological limitations need to be considered when interpreting the results. First, the assessment of trust and retirement intentions was based on self-report, which are subject to same source bias. Respondents with reduced physical and psychological well-being might be more likely to perceive and report low trust and be planning to leave the labor market, or more optimistic people may be more likely to perceive high trust and also have weaker retirement intentions. Respondents with early retirement intentions might also justify their aims by reporting poorer working conditions [11], [52]. However, this is not likely to explain our findings, since we used a prospective design, and the results persisted when focusing on participants with no baseline retirement intentions as well as when controlling for a large set of covariates such as health, health risk behaviors and dispositional optimism.

Second, the validity of our measure of trust can be questioned. Vertical and horizontal trust were each measured with only one single item, directly asking respondents to indicate their level of trust in their supervisor and in people at work, which may not capture all the variation in trusting relationships in the workplace. The wording in the two questions also differed somewhat from each other, and therefore they had slightly different emphasis. Inclusion of the word ‘really’ in the question of co-worker trust puts more emphasis on the respondent’s certainty of trust, while in the question of supervisor trust it was not included, which gives room to a certain degree of uncertainty. Earlier studies suggest that trust may be context-specific and depend on the situation, the task, and the person, and that surveys only gather a snap shot of trust at the time of data collection [49], [53]. On the other hand, Colquitt et al. [24] suggested that it may not matter in what sense one trusts; they did not find any significant differences in the associations between trust and its antecedents and consequences for different types of trust measures.

It is under debate whether trust can be dichotomized or whether it should be treated as a continuum. Studies on social capital tend to dichotomize trust (e.g. [33]), and we followed this literature. Other studies on trust in organizations often use trust as a continuous dimension (e.g. [31]).

Third, the study population consisted predominantly of female employees (77.4% women). Although the study population is representative of the Finnish public sector, the results might not be generalizable to other branches of industry or the working population in general, or to countries with different social security systems. Women may, for example, employ more collaborative behaviors to create a climate of trust in work teams than men [54]. In addition, in the public sector in Finland, women and men work in slightly different occupations [55] and may, therefore, differ in retirement attitudes [13], and men have also been shown to prolong their work career more often than women [27]. Studies have also found many similarities in retirement behavior across genders, and suggest that gender equality is increasing [56]. In Finland, the retirement age is the same for women and men [27], and there are no major gender differences in the participation in working life [55] and in the average effective age of labor market exit [29].

Finally, although we performed multiple adjustments, we cannot rule out the possibility that some unmeasured factors, such as job satisfaction or work motivation that were not available in the data, explain the observed associations between trust and retirement intentions.

### Conclusion

In this prospective study of municipal employees, we found that trust in the supervisor and trust in co-workers were associated with lower likelihood of retirement intentions. However, further research is needed to confirm the observed associations and to explore by how much supervisor and co-worker trust may extend working life. Further research is also needed to study possible mechanisms by which trust affects retirement intentions. Because several studies have suggested that retirement intentions predict actual early retirement [4], [8], our findings highlight the importance of trust in organizations and suggest that fostering trust in the workplace might have practical benefits. These observational findings suggest that increasing trust in the workplace may contribute to lengthening working careers and preventing early retirement.
